# Hypersaline sapropels act as hotspots for microbial dark matter

**DOI:** 10.1038/s41598-017-06232-w

**Published:** 2017-07-21

**Authors:** Adrian-Ştefan Andrei, Andreea Baricz, Michael Scott Robeson, Manuela Raluca Păuşan, Tudor Tămaş, Cecilia Chiriac, Edina Szekeres, Lucian Barbu-Tudoran, Erika Andrea Levei, Cristian Coman, Mircea Podar, Horia Leonard Banciu

**Affiliations:** 1 Department of Molecular Biology and Biotechnology, Faculty of Biology and Geology, Babeş-Bolyai University, Cluj-Napoca, Romania; 2 Institute of Hydrobiology, Department of Aquatic Microbial Ecology, Biology Center of the Academy of Sciences of the Czech Republic, České Budějovice, Czech Republic; 3 National Institute of Research and Development for Biological Sciences (NIRDBS), Institute of Biological Research, Cluj-Napoca, Romania; 4 Biosciences Division, Oak Ridge National Laboratory, Oak Ridge, Tennessee USA; 5 Interstitial Genomics, LLC, Longmont, 80501 Colorado USA; 6 Department for Internal Medicine, Medical University of Graz, Graz, Austria; 7 Department of Geology, Faculty of Biology and Geology, Babeş-Bolyai University, Cluj-Napoca, Romania; 8 INCDO-INOE 2000, Research Institute for Analytical Instrumentation, Cluj-Napoca, Romania

## Abstract

Present-day terrestrial analogue sites are crucial ground truth proxies for studying life in geochemical conditions close to those assumed to be present on early Earth or inferred to exist on other celestial bodies (e.g. Mars, Europa). Although hypersaline sapropels are border-of-life habitats with moderate occurrence, their microbiological and physicochemical characterization lags behind. Here, we study the diversity of life under low water activity by describing the prokaryotic communities from two disparate hypersaline sapropels (Transylvanian Basin, Romania) in relation to geochemical milieu and pore water chemistry, while inferring their role in carbon cycling by matching taxa to known taxon-specific biogeochemical functions. The polyphasic approach combined deep coverage SSU rRNA gene amplicon sequencing and bioinformatics with RT-qPCR and physicochemical investigations. We found that sapropels developed an analogous elemental milieu and harbored prokaryotes affiliated with fifty-nine phyla, among which the most abundant were *Proteobacteria*, *Bacteroidetes* and *Chloroflexi*. Containing thirty-two candidate divisions and possibly undocumented prokaryotic lineages, the hypersaline sapropels were found to accommodate one of the most diverse and novel ecosystems reported to date and may contribute to completing the phylogenetic branching of the tree of life.

## Introduction

Hypersaline ecosystems represent border-of-life habitats that are thought to be terrestrial analogue sites to Mars Meridiani Planum^1^ or Europa’s brine ocean^2^, and reasonable proxies for a Hadean-like origin-of-life environments^3^. Furthermore, with increasing evidence pointing out to the presence of briny liquid water on present-day Mars^4^ these space analogues became key ecosystems for understanding the adaptability of life (as we know it) at low water activity and for seeking potential microbial biosignature markers^5^. Despite the fact that recent studies on saline meromictic lakes^6^ and halite endoliths^7^ broadened our current view on microbial diversity and adaptability to the high-salt milieu, most of our current understanding of hypersaline environments is derived from studies performed on solar salterns, sabkhas or saline lakes. Although sapropels^8^ (i.e. unconsolidated, dark-coloured and organic carbon-rich lithologic layers that accumulate subaqueously in shallow to deep stagnant marine basins, lagoons and lakes^8^) generated in hypersaline lakes may be used as modern terrestrial analogues for Europan and past Martian environments, no in-depth microbiological and physicochemical analyses are available to date for these ecosystems.

These sediments enriched in organic matter found applicability in areas ranging from livestock farming and plant fertilizers to fuel industry and therapy^9^. As the increasing usage of sapropels significantly contributes to resource depletion, the need to characterize their microbiota rises out of necessity to replicate their natural formation process^10^ and to expand our knowledge on microbial diversification in energetically- constrained analogue environments. Although Romanian hypersaline sapropels have been used as cosmetics and medical peloids since the 19^th^ century to date^11^, their microbiological characterization is lacking as the few extant studies only dealt with enzymatic activity measurements or culturable diversity^12^. In this regard, the purpose of the present paper was to describe and link the prokaryotic diversity in two disparate hypersaline sapropels (Transylvanian Basin, Central Romania), with nutrient and geochemical data in order to infer the role of the microbiome in their formation, and to explore the microbial diversification at low water activity. To achieve these aims, prokaryotic communities were investigated by combining deep coverage SSU rRNA gene amplicon sequencing, community domain-specific quantitative PCR, physicochemical analysis and bioinformatics.

## Results and Discussion

The employed X-ray energy-dispersive and fluorescence methods (EDX and XRF) showed that the two sapropels harbored analogous elemental milieus (see Supplementary Table S1). The X-ray diffraction investigations (see Supplementary Fig. S1A) revealed the presence of similar mineralogical assemblages dominated by quartz, feldspars (plagioclase and/or orthoclase), muscovite and to a lesser extent, chlorite. The siliciclastic sapropels were found to contain high amounts of iron and silicon (Table 1, Supplementary Table S1), most likely entrapped within the crystal lattice of clay minerals (see Supplementary Fig. S1B), which could be released into the surrounding pore water by weathering or microbially-driven dissolution as shown by Vorhies & Gaines^13^. Moreover, high quantities of insoluble iron lacking clear diffraction patterns were detected in both environments and based on the *in situ* chemical milieu (Table 1), it was inferred that these ferric (oxyhydr-)oxides were generated by microbially-mediated anaerobic Fe^2+^ oxidation. It was considered that iron crystallization was hindered by its association with organic molecules, leading to the formation of stable organometallic structures within the anoxic environment^14^. Direct chelation or co-precipitation of organic carbon-iron structures^14^ coupled with a decreased rate of mineralization^15^, and the persistence of refractory organic molecules^16^ was assumed to be accountable for the preservation of organic carbon within these hypersaline sediments. Additionally, chemical analyses (Table 1) showed that dissolved electron acceptors (e.g. sulfate, nitrate, organic carbon) coexist with dissolved metabolic products (e.g. bicarbonate, sulfides, ammonium, methane), indicating that microbially-driven redox processes contribute to the degradation of the organic matter in these sapropels.

**Table 1:** Physicochemical and biological characteristics of the sapropels collected from Ursu and Fara Fund lakes (Transylvanian Basin, Central Romania) during October 2013.

Parameters	Lake	
	Ursu	Fara Fund
Depth (m)	3	2
ORP (mV)^I^	−120	−94
pH^I^	6.93	6.98
CH_4_ (mg/L)^a^	24	17
Total chlorophylls (µg/L)^I^	5412	357
Total carotenoids (µg/L)^I^	3852	468
Total cell counts/mL (×10^7^)^b^	3.3	2.9
Bacterial cell counts/mL (×10^8^)^c^	72.04	22.14
Archaeal cell counts/mL (×10^8^)^c^	10.65	2.28
Ammonium nitrogen (NNH_4_^+^, mg/kg)^II^	12.4	66.2
Nitrates (NO_3_^−^, mg/kg)^II^	0.75	6.7
Nitrites (NO_2_^−^, mg/kg)^II^	0.1	0
Organic nitrogen (ON, mg/kg)^II^	2687	3632
Total nitrogen (TN, mg/kg)^II^	2700	3700
Total carbon (TC, mg/kg)^III^	37000	12600
Total dissolved carbon (TDC, mg/kg)^II^	1410	6370
Dissolved organic carbon (DOC, mg/kg)^II^	1030	4900
Carbonate (CO_3_^2−^ as mg CaCO_3_ mg/kg)^II^	1400	1570
Phosphates (PO_4_^3−^, mg/kg)^II^	1320	14
Sulfates (SO_4_^2−^, mg/kg)^II^	140	767
Chlorides (Cl^−^, mg/kg)^II^	65100	41650
Bicarbonate (HCO_3_^−^, mg/L)^I^	1464	1450
Sulfides (S^2−^, mg/L)^I^	26	18.3
Potassium (K, mg/kg)^IV^	3690	2440
Iron (Fe, mg/kg)^IV^	24000	14300
Manganese (Mn, mg/kg)^IV^	1490	286
Calcium (Ca, mg/kg)^IV^	16000	4660
Magnesium (Mg, mg/kg)^IV^	5540	2720
Sodium (Na, mg/kg)^IV^	47000	31000
Total hydrolysable protein contents (%)	2.1	1.29
Moisture (%)	68.4	53.1
Average organic matter (OM %)^d^	4.72	4.64
Estimated total organic carbon (TOC %)^d^	2.36	2.32
Average total organic carbon (TOC %)^e^	4.2	1.8

^I^Measured from pore water. ^II^Measured as water-extractable compounds from dried sediments. ^III^Measured directly in the dried sediments. ^IV^Measured in dried sediments digested with aqua regia (HCl:NHO_3_ = 3:1). ^a^Measured at sediment-water interface. ^b^DAPI (4′,6-diamidino-2-phenylindole, dihydrochloride) cell counts. ^c^RT-qPCR (real-time quantitative PCR) cell counts. ^d^Estimation based on loss-on-ignition method. ^e^Estimation based on wet oxidation method.

Paired-end sequencing of 16 S rRNA gene hypervariable region V4 amplicons produced 147,227 high-quality reads (131,340 for Bacteria and 15,887 for Archaea) with an average length of 254 nt (see Supplementary Table S2). The Pearson’s correlation coefficient showed a positive strong correlation (r = 0.876) between the sequencing results and the real-time quantitative PCR data (Table 1). Furthermore, DAPI (4′,6-diamidino-2-phenylindole) epifluorescence microscopy revealed that sapropels were populated by small-sized (0.5–1 µm) bacilli, coccus-shaped prokaryotes and by larger (>1.5 µm) chlorophyll-containing microorganisms (see Supplementary Fig. S2). As expected, Bacteria dominated the sapropels (Table 1), harboring similar numbers with the ones reported for hypersaline sediments^17, 18^.

**Figure 1:**
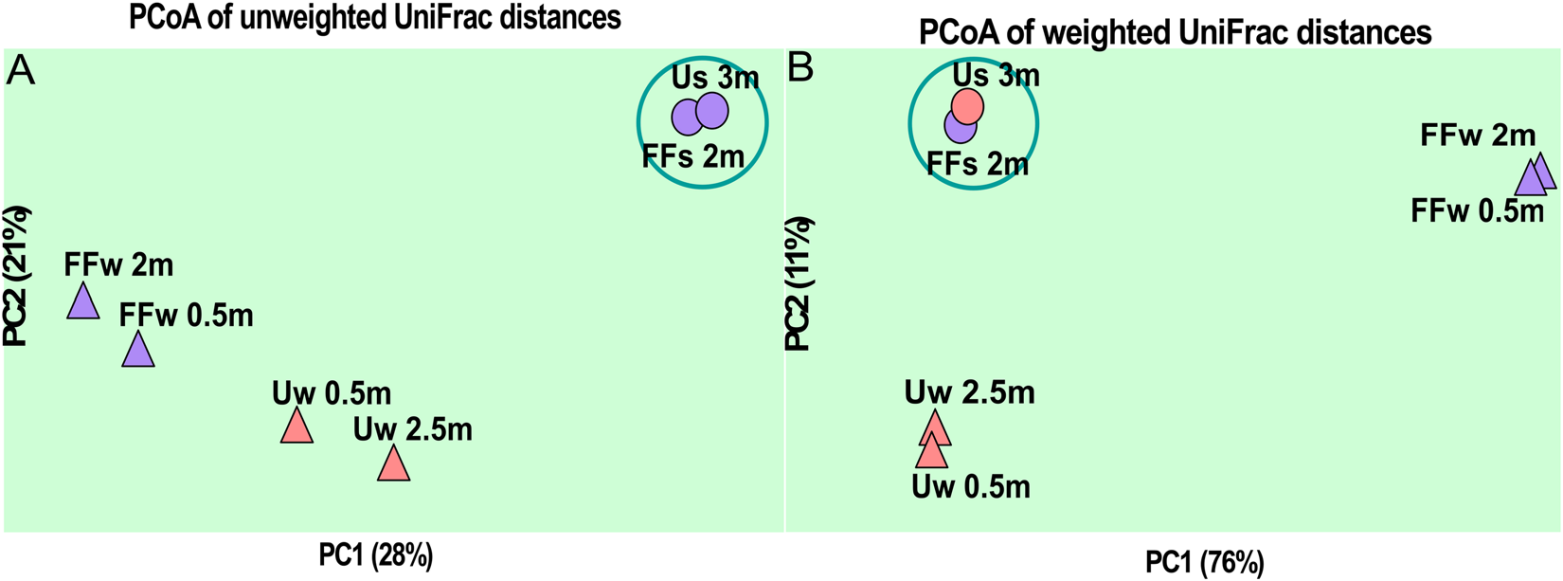
PCoA analyses of prokaryotic communities found in the water columns (triangles) and sapropels (circles) of Ursu and Fara Fund lakes, generated by using both unweighted (**A**) and weighted (**B**) UniFrac distance matrices. The water column sequences were recovered from SRA (accession numbers: SRS691458, SRS691457, SRS691436 and SRS691388). Abbreviations: Us3m – Ursu Lake sapropel sample from 3 m depth; FFs2m – Fara Fund Lake sapropel sample from 2 m depth; Uw0.5 m and Uw2.5 m – Ursu Lake water samples from 0.5 m and 2.5 m depths; FFw0.5 m and FFw2m – Fara Fund Lake water samples from 0.5 and 2 m depths.

**Figure 2:**
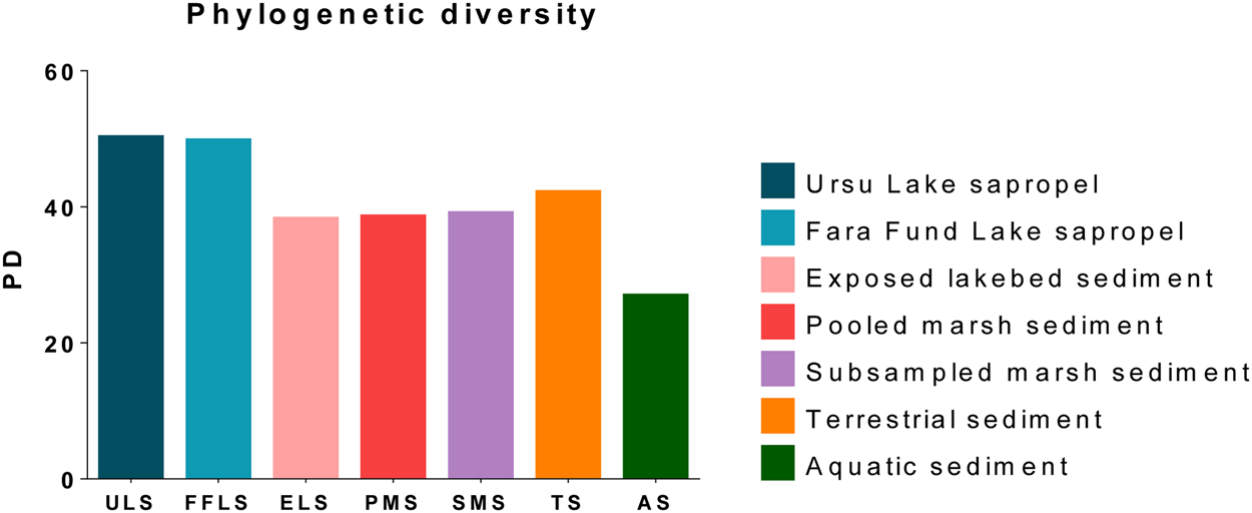
Phylogenetic diversity (Faith’s PD) estimates of Ursu (ULS) and Fara Fund (FFLS) sapropels in relation to other (hyper)saline sediments. The terrestrial (TS), aquatic (AS) and exposed lakebed (ELS) sediments are from La Sal del Rey hypersaline lake (sequences recovered from the following archives: SRS004880, SRS004879, SRS004878, SRS004877, SRS004876, SRS004875, SRS004874, SRS004873). The pooled (PMS) and subsampled marsh (SMS) sediments are from Rowley River salt marsh complex (sequences recovered from the following archives: SRS118669, SRS118662, SRS118663, SRS118664, SRS118665, SRS118666, SRS118667, SRS118668).

Beta-diversity analyses performed using sequences recovered from the water column (SRA accession numbers: SRS691458, SRS691457, SRS691436 and SRS691388) and sediments (generated by this study) showed that sapropels promote the formation of closely clustered idiosyncratic communities (Fig. 1). This grouping might be explained as the result of fundamental niche similarity (see physicochemical analyses) coupled with the phylogenetic and metabolic redundancy imposed by the high-salt environment^19^. Furthermore, the sapropels were found to harbor highly diverse and abundant microorganisms (Fig. 2, Table 1, see Supplementary Table S2) typical to (hyper)saline habitats (see Supplementary Fig. S3).

**Figure 3:**
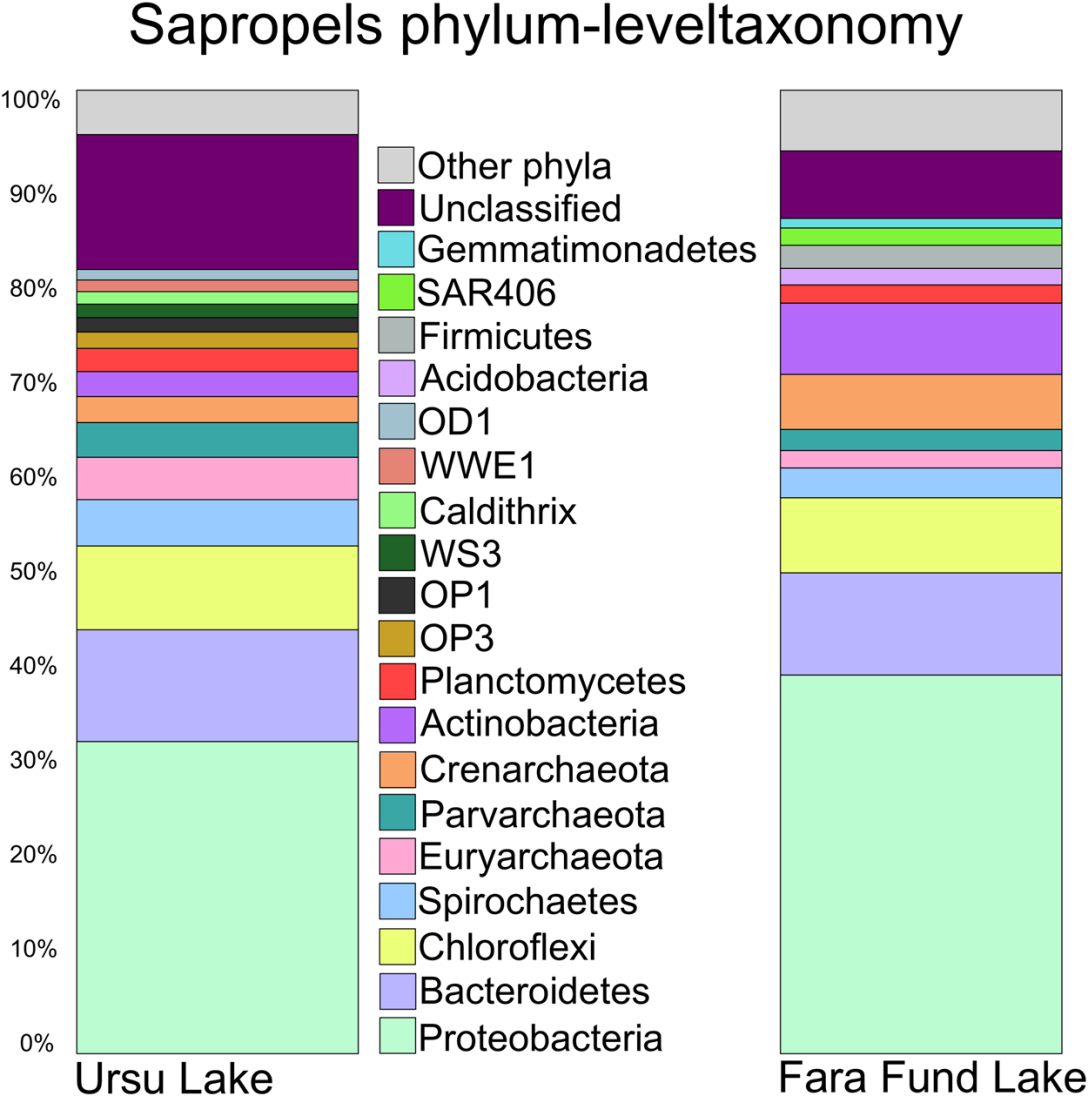
Phylum-level taxonomic profiles of sapropels prokaryotic communities using 16 S rRNA gene sequences.

Out of the total number of recovered sequences, ~6% did not classify in any bacterial phyla and were shown to be similar (87–100%) to uncategorized environmental sequences retrieved from habitats ranging from seabed sediments and hypersaline microbial mats to paddy soils and deep sea hypersaline basins (see Supplementary Table S3). We reason that a part of these unclassified sequences may represent undocumented prokaryotic lineages, indicating the presence of yet to be defined prokaryotic clades within microbial communities of sapropels (Fig. 3). The prokaryotic assemblages were dominated by *Proteobacteria* (~32 to ~39%), followed by *Bacteroidetes* (~11 to ~12%) and *Chloroflexi* (~8 to ~9%) (see Supplementary Table S4), which were found to be among the major phyla detected in salt marsh sediments^20^. Additionally, from the fifty-nine prokaryotic phyla detected, thirty-two were found to be candidate divisions with unknown cultivated representatives (i.e. “microbial dark matter” - MDM) and accounted remarkable abundances between ~8.3 and ~14.8% of the SSU rRNA gene sequences (Table S4). Within the MDM, the major phyla detected (≥1%) were Parvarchaeota (~2 to ~4%) and OP3 (~1 to ~2%) followed by OD1, WWE1, OP1, WS3 and SAR406 with ~1% abundances (Fig. 3).

We found that MDM phylogenetic enrichment in the hypersaline sapropels was unprecedented and that the microbial phylogenetic diversity (Fig. 2) was greater than the one previously described in the highly diverse hypersaline sediments^20, 21^. This high level of phylogenetic diversity (see Supplementary Table S2) may be attributed to the habitat diversification triggered by downward metabolite fluxes^22^ and to the high variety of energetic pathways found in sapropels that may lower the interspecific competition.

**Figure 4:**
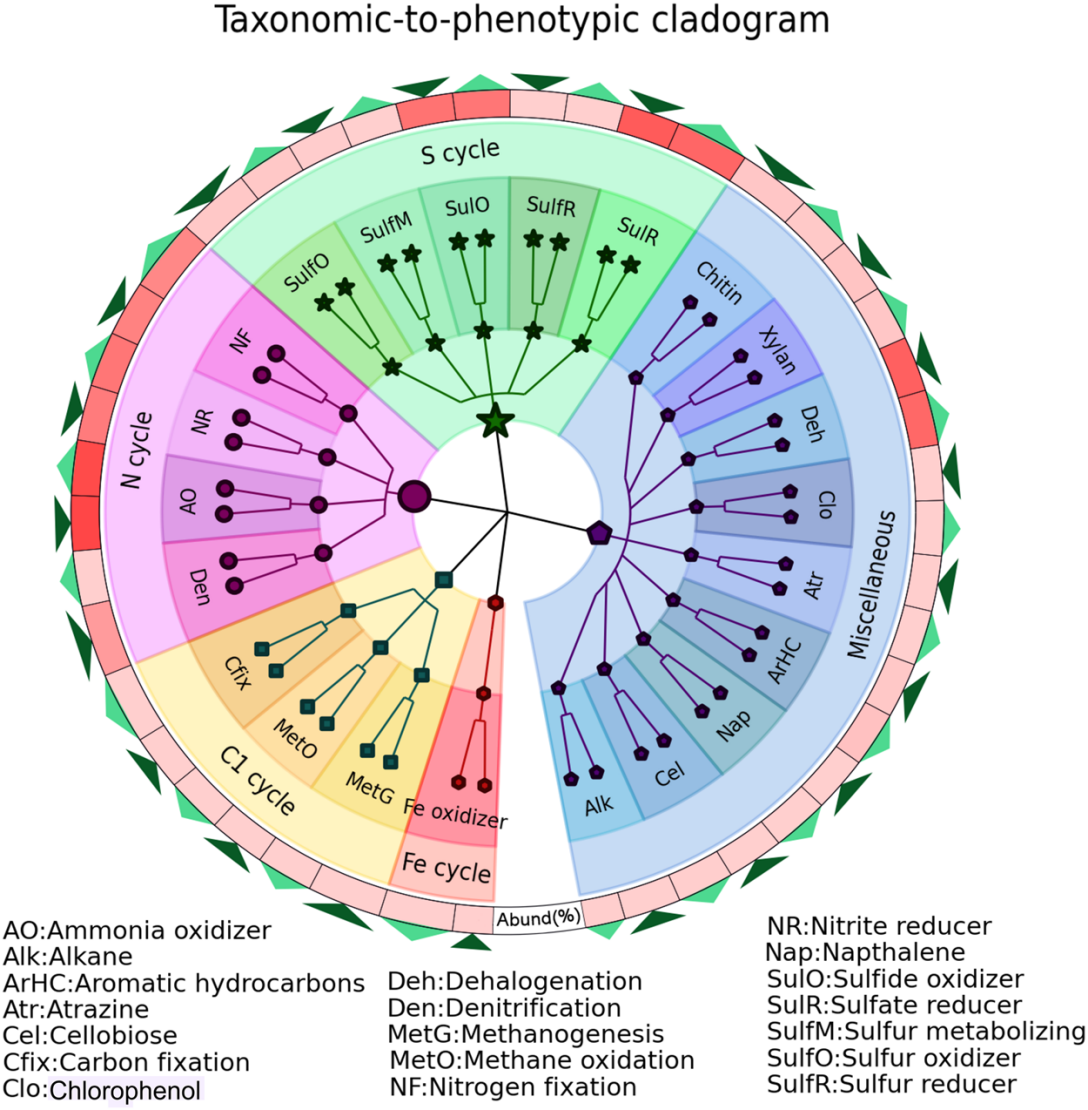
Sapropels’ taxonomic-to-phenotipic cladogram showing the putative metabolic profiles of sapropels’ microbial communities (based on 16 S rRNA gene). The cladogram does not reflect the functional status of the microbial communities, but rather their metabolic potential. The red internal ring is a circular heatmap; the colour intensity is proportional with the number of sequences affiliated with a metabolic profile. The emerald triangles (▴) correspond to the metabolic profiles of Ursu sapropels, while the inverted (▾) green ones correspond to Fara Fund sapropels.

By matching taxa to known taxon-specific biogeochemical functions (Fig. 4), we assume that organic carbon is predominantly mineralized in anaerobic food webs (formed by prokaryotes and fungi, see Supplementary Figs S4 and S5) in which sulfate reduction is probably the major mechanism involved in its oxidation. We consider that sapropels are enriched with MDM due to its capacity to anaerobically utilize refractory substrates (e.g. OD1 and WWE1), establish syntrophic networks (e.g. OP1) or generate energy by linking the sulfur and iron cycles (e.g. WS3, OP3 and SAR406)^23–28^. Nevertheless, more data is needed to pinpoint the roles of these uncultured prokaryotic clades within microbial communities. Furthermore, by considering that the used DNA-based methods reflect both the metabolically active and inactive cells, and that extracellular DNA has the capacity to adsorb to negatively charged particles (e.g. silica, clay, organic matter) via phosphates and cation bridging^29^, we assume that the phylogenetic profiles emulate the sapropels diversity potential. We underline the fact that the described diversity was composed by prokaryotes actively living in the sapropels and also by the ones that contributed to the extracellular DNA pool^30^. Although sapropels were collected from lakes with a highly dissimilar water column microbiota and located more than 100 km away^6^ (Fig. 1), they harboured analogous microbial communities (Fig. 1) indicating a habitat- specific microbiome that was selected by the distinct physicochemical milieu and which did not originate in the water column. Moreover, recent data on soil microbiome highlighted that extracellular DNA closely reflects the taxonomic composition of microbial communities^31^.

Overall, the present paper describes the biogeochemical baseline of hypersaline sapropels and at the same time represents the first attempt to use deep amplicon sequencing in surveying their microbial diversity. By using a polyphasic approach, we found that sapropels harbor one of the most diverse and novel microbiota reported to date, and probably encompass novel prokaryotic clades. Furthermore, we reason that these extreme environments contain an unforeseen amount of MDM phylogenetic diversity and could further serve as a framework for targeting the metabolic potential of these elusive microorganisms.

As in the current view, the hypersaline environments (*sensu lato*) are regarded as habitats with limited microbial diversity and terrestrial analogues to Mars and Europa, we postulate that a paradigm change is necessary to accommodate our results and shed light on the links between chemical and biological diversity, all the more as our quest for extraterrestrial biosignatures is higher than ever before.

## Methods

### Site description and sampling

Ursu (46°36′15 N; 25°05′09 E) and Fara Fund (45°52′34 N; 24°04′03 E) are two hypersaline meromictic lakes (situated at 112.6 km apart) located in the Transylvanian Basin (Romania), that formed on the salt deposits generated during the Paratethys Sea evaporation (ca. 14 Ma ago). Having a surface of 41,270 m^2^ and lying in the eastern part of the basin, Ursu Lake formed between 1875–1880 after a catastrophic event caused by an intense process of salt dissolution^32^. With a surface of 1,700 m^2^ and situated in the southern part of the basin, Fara Fund Lake was formed in 1775 following the flooding of a late medieval bell-shaped salt mine^32^. The limnological characteristics of the lakes’ water columns and their microbiota were recently described by Andrei *et al*.^6^.

Sapropel samples were collected in October 2013 from the euphotic zone of the lakes (i.e. 3 m depth in Ursu Lake and 2 m depth in Fara Fund Lake) using a Petite Ponar dredge (Wildco, Saginaw, MI, USA) handled from an inflatable boat. *In situ* measurements (e.g. pH, conductivity and redox potential) were performed using a portable water multiparameter system HI 9828 (Hanna Instruments, Woonsocket, RI, USA). The sapropel samples were deposited in sterile 1 L polypropylene bottles, at 4 °C, and transported within 6 hours to the laboratory, where they were immediately stored at −20 °C until DNA extraction.

### Environmental DNA (eDNA) extraction and real-time quantitative PCR (RT-qPCR)

Sapropel samples (0.5 g) were processed for DNA extraction using the ZR Soil Microbe DNA MiniPrep kit (Zymo Research, Irvine, CA, USA) and the PowerSoil DNA isolation kit (MoBio Laboratories, Carlsbad, CA, USA), following the manufacturer’s instructions. The DNA was extracted in duplicate from each sample and stored at −20 °C until further use. The isolated eDNA reflected the total pool of DNA harbored by the sapropels at time of sampling, and contained both intracellular (found in living, dormant or dead microbiota) and extracellular DNA (originated from exudation/excretion from viable cells and cellular (auto)lysis).

The absolute quantification of archaeal and bacterial 16 S rRNA gene copies was performed using the SsoFast Eva Green Supermix (Bio-Rad, Hercules, CA, USA) and the Archaea 931F/M1100R^33^ and Bacteria 338F/518R^34, 35^ primer sets on an iCycler IQ5 Real-Time System (Bio-Rad, Hercules, CA, USA). For archaeal 16 S rRNA gene amplification reactions were carried out in triplicate and the reaction mixture contained the following components: 7 μl 1 × Sso Fast EvaGreen SuperMix (Bio-Rad, Hercules, CA, USA), 0.4 μM of the forward 931F and reverse M1100R primers, 10 ng of DNA and RNase/DNase-free water to a final volume of 14 μl. The reactions were carried out as follows: 180 s initial denaturation at 98 °C, followed by 45 cycles of: 25 s denaturation at 98 °C, 25 s primer annealing at 61.5 °C, and 30 s extension at 72 °C. For assessing the specificity of the primers, a post-PCR melting curve analysis was performed, in which the temperature varied between 60 °C and 90 °C in 0.5 °C increments with subsequent plate readings. The same reaction components and protocol were used for the bacterial RT-qPCR with the following modifications: the primers used were 338F and 518R, and their annealing temperature was 61 °C. Reaction efficiencies and data analyses were assessed using the background subtracted data and the LinRegPCR software. The numbers of cells from the numbers of 16 S rRNA genes found in the samples was inferred taking into account the variation in ribosomal RNA operons (rrnDB version 4.3.3).

### Amplicon sequencing and bioinformatics analyses

The sapropel data generated by this study were prepared as per Andrei *et al*.^6^ and trimmed to 254 bp. Briefly, the molecular tagging and amplification reactions followed the protocol of Lundberg *et al*.^36^. For each sample three independent PCRs, targeting the hypervariable region V4 of the 16 S rRNA gene, were combined in equimolar rations and used in metabarcoding library construction. Sequencing of bacterial and archaeal V4 amplicons was performed on the Illumina MiSeq platform (San Diego, CA, USA) at the Oak Ridge National Laboratory (Oak Ridge, TN, USA). Sequence data were processed and quality controlled through a combination of the UPARSE and QIIME pipelines^37, 38^. Cutadapt^39^ was used in ‘paired-end mode’ to trim sequencing primers from the forward and reverse reads while simultaneously discarding those read pairs in which both the forward and reverse primers were not detected (allowed for 10% mismatches for primer search). Paired-ends were merged using the -fastq_mergepairs option of usearch^37^. An in-house python script was used to remove unused barcodes of paired-end sequences that did not survive merging. Furthermore, the QIIME (script, split_libraries_fastq.py, was used to demultiplex the sequence data with the quality filter set to zero. Quality filtering was performed within usearch (UPARSE).

Sapropel community data was compared to other communities within similar hypersaline environments using closed-reference OTU picking *via* QIIME. The closed-reference approach limits our ability to detect novel community sequence diversity by retaining only those sequences that match full-length curated (chimera-free) sequences contained within the Greengenes reference database^40, 41^. Thus, if a given sequence did not have at least a 97% similarity with any of the curated sequences within the Greengenes database, that sequence, especially if chimeric, was discarded. Any chimeras that are 97% similar to an existing GreenGenes reference OTU will simply be subsumed into that OTU. However, chimeras are typically far more divergent than 3% and is one of the main assumptions of many OTU-picking and chimera removal tools, e.g. UCHIME and UPARSE. Thus, the presence or absence of OTUs are limited to what exists within the GreenGenes reference OTU database. This allows us to compare our data with those of other studies as they will be condensed to a common OTU reference space (Navas-Molina *et al*., 2013). The previously published data sets of Hollister *et al*.^21^ and Bowen *et al*.^20^ were downloaded from the GenBank SRA (SRS004880, SRS004879, SRS004878, SRS004877, SRS004876, SRS004875, SRS004874, SRS004873, SRS118669, SRS118662, SRS118663, SRS118664, SRS118665, SRS118666, SRS118667, SRS118668) and used for phylogenetic diversity (i.e. Faith’s phylogenetic diversity) estimations. Sequence data was processed as above by QIIME and usearch.

Due to the different nature in which both the Hollister *et al*.^21^ and Bowen *et al*.^20^ data were sequenced, reads from each of these studies were trimmed to 250 and 54 bp respectively. Then, closed-reference OTU-picking was performed by using SortMeRNA^42^ at 97% sequence similarity to the 13_8 Greengenes database^41^ *via* the script ‘pick_closed_reference_otus.py’ from QIIME. The closed-reference OTU tables were then merged with the QIIME script ‘merge_otu_tables.py’. All down-stream phylogenetic diversity analyses were performed in QIIME by rarefying the data to 1225 reads per sample. We have observed no noticeable differences in our results when trimming all the sequence data to 54 bp (smallest read length of all the data) prior to closed-reference OTU-picking compared to using all the available read data for each study. Taxonomic-to-phenotypic mapping was performed by linking OTUs nomenclature to taxonomic assignments in METAGENassist^43^. The graphical representation of the phenotipic cladogram was constructed with GraPhlAn software^44^.

### Nucleotide sequence accession numbers

All sequence data are available through the National Center for Biotechnology Information (NCBI) under the accession numbers: SRR2043654 and SRR2043661 (sequences generated by the metabarcoding study) and KR610415-KR610425 (fungal sequences).

## Electronic supplementary material

Supplementary Information
