# Supplementary material for: Hypersaline sapropels act as hotspots for microbial dark matter
Source: Sci Rep. 2017 Jul 21;7:6150. doi: 10.1038/s41598-017-06232-w (PMC5522462; doi:10.1038/s41598-017-06232-w)

Supplementary Information

Hypersaline sapropels act as hotspots for microbial dark matter

Adrian-Ştefan Andrei[1,2*], Andreea Baricz[3], Michael S. Robeson II[4,5], Manuela Raluca Păuşan[6], Tudor Tămaş[7], Cecilia Chiriac[3], Edina Szekeres[3], Lucian Barbu-Tudoran[1], Erika Andrea Levei[8], Cristian Coman[3], Mircea Podar[4,] and Horia Leonard Banciu[1*]

Department of Molecular Biology and Biotechnology, Faculty of Biology and Geology, Babeş-Bolyai University, Cluj-Napoca, Romania[1]; Institute of Hydrobiology, Department of Aquatic Microbial Ecology, Biology Center of the Academy of Sciences of the Czech Republic, České Budějovice, Czechia[2]; National Institute of Research and Development for Biological Sciences (NIRDBS), Institute of Biological Research, Cluj-Napoca, Romania[3]; Biosciences Division, Oak Ridge National Laboratory, Oak Ridge, Tennessee, USA[4]; Interstitial Genomics, LLC,Longmont, Colorado, USA[5]; Department for Internal Medicine, Medical University of Graz, Graz, Austria[6]; Department of Geology, Faculty of Biology and Geology, Babeş-Bolyai University, Cluj-Napoca, Romania[7]; INCDO-INOE 2000, Research Institute for Analytical Instrumentation, Cluj-Napoca, Romania[8].

[*]**Corresponding authors:** Department of Molecular Biology and Biotechnology, Babeş-Bolyai University, 5-7 Clinicilor Str., 400006 Cluj-Napoca, Romania. Telephone: +40-264431691; fax: +40-264431858. E-mail: stefan.andrei @ubbcluj.ro; horia.banciu@ubbcluj.ro.

Supplementary methods

Analytical techniques

The extraction and estimation of dissolved CH_4 concentrations at the sediment-water interface were determined at the sampling site, using the head-space method on an instrumental package (West System, Pontedera, Italy) equipped with semiconductor (range 0-2000 ppmv; lower detection limit of 1 ppmv; resolution 1 ppmv), catalytic (range 2000 ppmv – 3 % v/v) and thermal conductivity (range 3 % - 100 % v/v) detectors.

The organic matter content (% OM) of sapropel samples was determined by using the loss-on-ignition (LOI) and wet oxidation techniques. LOI determination was executed as described by Ball[1], with the assumption that the total organic carbon content (% TOC) is 50 % of the OM. The dichromate oxidation followed by titration was performed as described by Walkley and Black[2], following a % TOC correction estimation by a factor of 1.16 (equivalent to ca. 86 % organic carbon recovery).

As most of the organic nitrogen in aquatic sediments seems to be deposited under the form of amide bonds[3], the total alkaline extractable protein content was estimated in the sapropels by the Lowry method[4] modified by Rice[5]. Ammonium nitrogen was quantified by colorimetry using a Lambda 25 spectrophotometer (Perkin Elmer, Beaconsfield, UK). The contents of total nitrogen (TN) as bound nitrogen (including free ammonia, ammonium, nitrite, nitrate and organic nitrogen, but not dissolved nitrogen gas) were assessed by thermal oxidation, conversion of nitrogen oxides to NO, followed by NO oxidation with ozone and subsequent chemiluminescence detection using the Multi N/C 2100 S Analyzer (Analytik Jena, Jena, Germany). Organic nitrogen (ON) in the form of dissolved organic nitrogen was calculated by subtracting ammonium nitrogen, nitrate and nitrite nitrogen from the total nitrogen. Total carbon (TC), total dissolved carbon (TDC) and dissolved organic carbon (DOC - calculated as difference between total dissolved carbon and dissolved inorganic carbon measured upon sample acidification) were quantified by combustion and NDIR detection of CO_2 released using the multi N/C 2100 S Analyzer. Chloride (Cl^-), carbonate (as $CaCO_3$) and bicarbonate (HCO_3^-) anions were measured by titrimetric methods. Phosphate (PO_4^{3-}), sulfate (SO_4^{2-}), nitrate (NO_3^-), and nitrite (NO_2^-) were measured by ion chromatography on a 761 Compact IC (Metrohm, Herisau, Switzerland). The concentration of sulfides was determined by methylene blue method after fixation of samples with 2 % (v/v) Zn-acetate[6]. Concentrations of major metals (Mg, Ca, Na, K, Fe, Mn) were measured by inductively coupled plasma atomic emission spectrometry

(ICP-AES) using OPTIMA 5300 DV spectrometer (Perkin Elmer, Norwalk, USA) after homogenization of sediments and digestion in concentrated *aqua regia*. Relative expanded measurement uncertainty ($U_{Erel.}$) was calculated according to ISO-GUM using a coverage factor (k) of 2 (k = 2), equivalent to a confidence of approximately 95 %. Uc ranged from 4.5 to 13 % depending on the compound as follows: 8 % for TC, HCO_3^-, Fe, Mn, Mg and Ca; 9.5 % for PO_4^{3-}; 9.8 % for Na^+; 10 % for TN, TDC, CO_3^{2-}, SO_4^{2-}, HS^- and K; 11 % for NO_3^- and ON; 13 % for NO_2^-, NH_4^+ and Cl^-.

For X-ray fluorescence analysis (XRF), sapropel samples were dried to constant weight at 60 °C and grinded to pass a < 200 µm sieve. Major and trace elements (Cl, Fe, Ca, K, Ti, S, Mn, Cu, Zn, etc.) were determined by using a dispersive X-ray fluorescence spectrometer (Innov-X Alpha 6500, The Netherlands) equipped with X-ray tube, W anode and Si PiN diode detector. XRF spectra were recorded at Kα line on samples placed in small plastic cups covered by Mylar X-ray window film. The energy-dispersive X-ray (EDX) spectroscopic analyses of dried sapropel samples were performed over an area of 250 x 200 μm^2, at 20 keV beam energy using a FEI Quanta 3D FEG dual beam SEM equipped with an energy dispersive X-ray detector.

The mineral composition of bulk sediment samples was assessed by X-ray diffraction (XRD), using a Bruker D8 Advance diffractometer with $CoK\alpha_1$ with λ = 1.78897, Fe filter and a one-dimensional detector using corundum (NIST SRM1976a) as internal standard. The data were collected on a 5 - 64° 2θ interval, at a 0.02^o 2θ, with the measuring step of 0.2 seconds. The clay fraction was analyzed on oriented samples (pipetted from suspension in distilled water), which were further treated with ethylene glycol. Data were collected on the same interval as mentioned above, with a measuring step of 0.5 seconds. The identification of mineral phases was performed with the Diffrac.Eva 2.1 software from Bruker AXS, using the PDF2 (2012) database.

SEM, cell counts, chlorophyll *a* and total carotenoids analyses

For SEM analyses, samples of sapropel and plant material recovered from sapropels were fixed in 4 % glutaraldehyde, filtered onto 0.25 µm polycarbonate Millipore filters (Bedford, MA, USA) and subsequently washed with 1 x PBS (pH 7.4). Henceforth, they were subjected to an alcohol dehydration series after which they were fixed with 100 % hexamethyldisilizane (Electron Microscopy Sciences, Hatfield, Pennsylvania) and air-dried. After mounting on SEM specimen holders they were sputter-coated (Agar Automatic Sputter Coater, Agar Scientific, UK) with 7-10 nm of gold and visualized with a Jeol JEM 5510LV scanning electron microscope operated at 15 kV. For total cell counts, sapropel pore water samples were fixed in 1 % glutaraldehyde

and filtered through black, gridded cellulose ester membrane filters (0.45 µm, d=47 mm). Subsequently, they were stained directly using 5 µg/mL of DAPI (4',6-diamidino-2-phenylindole, dihydrochloride) solution and examined by epifluorescence (BX60, Olympus Optical, Tokyo, Japan). Chlorophyll *a* and total carotenoids concentrations were determined as described by Wetzel and Likens[7].

Fungal community analyses

The collected sapropel samples were subsampled (i.e. 3 subsamples from each sapropel sample) and small pices of decaying plant material (leaves) were recovered from Ursu and Fara Fund and processed for DNA extraction using the ZR Soil Microbe DNA MiniPrep kit (Zymo Research) in accordance with the manufacturer's instructions. The PCR reactions were carried out in triplicates using the primers pair FR1-GC/FF390 as described by Vainio and Hantula[8]. The purified PCR-amplified DNA products were compared (700 ng) by DGGE analysis on 8 % (w/v) polyacrylamide gels (containing 37.5:1 of acrylamide to bisacrylamide) with a linear denaturing gradient ranging from 40 % to 65 % (the 100 % denaturant contains 40 % v/v formamide and 7 M urea). Gels electrophoresis were run for 18 h at a constant temperature (58 °C) and voltage (50 V) in 1% Tris-acetate-EDTA buffer, using the DCode™ Universal Mutation Detection System (Bio-Rad, Hercules, CA, USA). Subsequently, the gels were stained with a 1 x SYBR Gold® solution (Invitrogen, Carlsbad, CA) for 40 minutes and visualized using the UVP Imaging and Analysis System and LabWorks software (UVP Inc.). The most intense DGGE bands were excised and incubated overnight in ultrapure water at 4 °C. 3 µl of the DNA-containing water was used in PCR reactions and the purified products were cloned using a commercial system (InsTAclone™ PCR cloning kit, Thermo Scientific) according to the manufacturer's instructions. Plasmids were purified using the GeneJet Plasmid Miniprep kit (Thermo Scientific) and sequenced using the Sanger method at a commercial company (Macrogen Europe). The fungal community DGGE fingerprints were analyzed with the software package BioNumerics 6.5 (Applied Maths, Ghent, Belgium) according to the provider's instructions.

References

1. Ball, D.F. Loss-on-ignition as an estimate of organic matter and organic carbon in non-calcareous soils. *J. Soil Sci*. **15**, 84-92 (1964).

2. Walkley, A. & Black, I.A. 1934. An examination of the Degtjareff method for determining soil organic matter, and a proposed modification of the chromic acid titration method. *Soil Sci*. **37**,

29-38 (1934)

3. Fernandes, L., Garg, A. & Borole D.V. Amino acid biogeochemistry and bacterial contribution to sediment organic matter along the western margin of the Bay of Bengal. *Deep Sea Res. Part I: Oceanogr. Res. Papers* **83**, 81-92 (2014).

4. Lowry, O.H., Rosebrough, N.J., Farr, A.L. & Randall, R.J. Protein measurement with the Folin phenol reagent. *J. Biol. Chem.* **193**, 265-275 (1951).

5. Rice, D.L. The detritus nitrogen problem: new observations and perspectives from organic geochemistry. *Mar. Ecol. Prog. Ser*. **9**, 153-162 (1982).

6. Trüper, H.G. Schlegel, H.G. Sulfur metabolism in Thiorhodaceae. 1. Quantitative measurements on growing cells of *Chromatium okenii*. *Antonie van Leeuwenhoek* **30,** 225–238 (1964).

7. Wetzel, R.G. & Likens, G.E. *Limnological Analyses* 39 (Springer-Verlag, New York, 1991)

8. Vainio, E.J. & Hantula, J. Direct analysis of wood-inhabiting fungi using denaturing gradient gel electrophoresis of amplified ribosomal DNA. *Mycol. Res*. **104**, 927-936 (2000).

Supplementary tables

Table S1. Semiquantitative analysis of elements in dried sapropel samples from Ursu and Fara Fund lakes by X-ray fluorescence (XRF) and scanning-electron microscopy/energy-dispersive X-ray (SEM/EDX). Values are average of three measurements in XRF and five different scanning of distinct spot areas in SEM/EDX.

Element	Element concentration (%dw)			
	Ursu Lake sediment		Fara Fund Lake sediment	
	XRF	EDX	XRF	EDX
	Major elements (>1 %)			
**C**	NA	6.8	NA	5.3
**O**	NA	37.8	NA	35.2
**Si**	NA	21.8	NA	28.4
**Na**	NA	5.3	NA	3.7
**Al**	NA	6.2	NA	7.3
**Cl**	4.4	6.6	2.9	4.3
**Fe**	3.2	2.3	2.2	2.1
**Ca**	1.9	1.2	1.0	0.8
**K**	1.1	1.2	1.8	1.3
	Minor elements (0.1-1 %)			
**Mg**	NA	0.8	NA	0.6
**Ti**	0.44	0.3	0.45	0.5
**S**	0.13	0.6	0.10	0.5
**Mn**	0.15	0.2	0.04	0.1
	Trace elements (<0.1 %)			
**Ba**	0.022	ND	0.019	ND
**Zr**	0.014	ND	0.020	ND
**Sr**	0.013	ND	0.014	ND
**Cu**	0.010	Traces	0.010	Traces
**Zn**	0.011	ND	0.006	ND
**Rb**	0.007	ND	0.005	ND
**Co**	0.005	Traces	<0.002	Traces
**Br**	0.001	Traces	<0.0007	Traces
**Pb**	0.002	Traces	0.002	Traces
	Elements below detection limits of XRF			
**Ni, Cr, As, Se, Mo*, Ag, Cd, Sn, Sb, I, Au, Hg**				

* Traces of Mo were observed by EDX analysis.
Abbreviations: NA – not analyzed; ND – not detected.

Table S2. Total read counts and alpha diversity estimates for the sapropels from Ursu and Fara Fund lakes

	**Ursu Lake**	**Fara Fund Lake**
**Total read counts**	84,125	63,102
**Number of OTUs per sample**	2,121	1,806
**Good's coverage**	0.992	0.985
**Phylogenetic diversity (PD)**	113.731	93.981
**Simpson (S)**	0.996	0.995
**Shannon (H)**	9.444	9.053

Table S3. Nearest BLAST hits of the archaeal and bacterial OTUs classified within the uncultivated and candidate bacterial and archaeal phyla. Operational taxonomic units are organized by decreasing abundance of each phylum as well as decreasing abundance of each OTU within the respective phylum.

A. Ursu Lake

OTU*	Classification	Sequence number	Nearest match (% identity)	Source location of the match	GenBank ID
192	Bacteria	118	Clone MEDEE_16SB_65_MIF (98 %)	Hypersaline Lake Medee, Eastern Mediterranean	JX446064.1
388	Bacteria	88	Clone MS-A229 (87 %)	Calcareous sandy sediment, Spain	FJ949402.1
465	Bacteria	88	Clone MEDEE_16SB_542_MIF (98 %)	Hypersaline Lake Medee, Eastern Mediterranean	JX446043.1
421	Bacteria	87	Clone C149 (99 %)	Soil, China	KJ817694.1
411	Bacteria	84	Clone B0618R002_D01 (96 %)	Rice paddy soil, Japan	AB658668.1
190	Bacteria	82	Clone GUAY_50enr_Bac50 (94 %)	Hydrothermal sediments, Guaymas Basin, Pacific Ocean	FR682656.1
284	Bacteria	80	Clone: p816_b_5.05 (91 %)	Sediment, hydrothermal vent, Okinawa Trough	AB305603.1
573	Bacteria	75	Clone GUAY_37enr_Bac5 (89 %)	Hydrothermal sediments, Guaymas Basin, Pacific Ocean	FR682626.1
436	Bacteria	71	Clone Shimokita-2-70 (89 %)	Deep-sea surface sediment, Shimokita, Pacific Ocean	AB734635.1
538	Bacteria	63	Clone TKTMmva-B45 (88 %)	Hydrothermal vent, Okinawa, Japan	AB611531.1
691	Bacteria	63	Clone Exp331_INH_50U_23 (97 %)	Sediment, Okinawa Trough, Japan	AB825411.1
4027	Bacteria	56	Clone MEDEE_16SB_542_MIF (96 %)	Hypersaline Lake Medee, Eastern Mediterranean	JX446043.1
707	Bacteria	48	Clone pYK04-20B-60 (99 %)	Hydrothermal vent, Okinawa, Japan	AB464834.1
409	Bacteria	45	Clone MAT-CR-H3-H11 (97 %)	Hypersaline microbial mat, Candeleria lagoon, Puerto Rico	EU245196.1
694	Bacteria	44	Clone N2_D1 (100 %)	Microbial mat petola, Slovenia	FN678681.2
934	Bacteria	44	Clone Pre-sep_090802W1 (87 %)	Hot spring, USA	KM697417.1
976	Bacteria	44	Clone MEDEE_16SB_42_MIF (98 %)	Hypersaline Lake Medee, Eastern Mediterranean	JX446051.1
571	Bacteria	43	Clone SBYZ_1565 (94 %)	Hypersaline mat, Guerrero Negro, Mexico	JN496253.1
85	OP1	878	Clone 15-P2 (97 %)	Hypersaline sediment, Death Valley, USA	AB533929.1
124	OP1	143	Bacterium Medee_0001 (100 %)	Brine, Medee Lake, Eastern Mediterranean	JX454600.1
102	OP1	87	Clone G06U47 (99 %)	Evaporitic crust, Guerrero Negro, Mexico	EF106490.1
417	OP1	46	Clone GeoB15101-6-B5-Bact-93 (96 %)	Urania Basin brine, Eastern Mediterranean	KM278921.1
179	OP3	626	Clone MEDEE_16SB_09_UIF (98 %)	Water, Hypersaline Lake Medee, Eastern Mediterranean	JX446112.1
676	OP3	62	Clone p816_b_5.05 (87 %)	Hydrothermal sediments, Southern Okinawa Trough, Japan	AB305603.1
654	OP3	54	Clone E6aB08 (100 %)	Hypersaline endoevaporitic microbial mat, Eilat, Israel	DQ103667.1
728	OP3	50	Clone 113B509 (93 %)	Sediment - Chefren mud volcano, Eastern Mediterranean	EF687513.1
880	OP3	43	Clone WLS4-5 (98 %)	Wuliangsuhai Lake, China	KC454612.1
970	OP3	43	Clone B41.20 (95 %)	Sediments, Bohai Sea	KF856854.1
278	WS3	318	Clone SBZP_6337 (100 %)	Hypersaline mat, Guerrero Negro, Mexico	JN539471.1
137	WS3	206	Clone HA9-SRB-CD3 (97 %)	SedimenT, hydrothermal vent, Bransfield Strait, Antarctica	FM868270.1
288	WS3	170	Clone SBZI_8025 (99 %)	Hypersaline mat, Guerrero Negro, Mexico	JN526162.1
535	WS3	68	Clone PM-150-Bact-24 (96 %)	Sediment, cold seep, Pakistan Margin	JQ925060.1
754	WS3	46	Clone V1F127b (97 %)	Iron oxide sediments, Volcano 1, Tonga Arc	FJ905749.1
1043	WS3	44	Clone SBZC_5732 (100 %)	Hypersaline mat, Guerrero Negro, Mexico	JN509263.1
49	WWE1	508	Clone 104B375 (92 %)	Sediment, Chefren mud volcano, Eastern Mediterranean	EF687304.1

340	WWE1	140	Clone E23 (94 %)	Brackish sediments, Bouches-du-Rhone, France	JQ771472.1
534	WWE1	114	Clone PM-150-Bact-24 (96 %)	Sediment - cold seep, Pakistan Margin	JQ925060.1
823	WWE1	70	Desulfohalobium retbaense strain DSM 5692 (100 %)	Saline sediment - Lake Retba, Africa	NR_074907.1
302	Parvarchaeota	161	Clone OGAP_AB16_38cm_06 (91 %)	Sediments, West Pacific abyssal plain, Japan	AB827099.1
426	Parvarchaeota	113	Clone GN28DB130 (99 %)	Hypersaline mat, Guerrero Negro, Mexico	EU731128.1
536	Parvarchaeota	101	Clone OGAP_AB16_38cm_27 (93 %)	Sediments from West Pacific abyssal plain, Japan	AB827103.1
548	Parvarchaeota	62	Clone AS07-7-39 (83 %)	Biofilm from subsurface sulfidic cave stream	KM410335.1
826	Parvarchaeota	59	Clone SBZP_6687 (97 %)	Hypersaline mat, Guerrero Negro, Mexico	JN539783.1
704	Parvarchaeota	58	Clone smkt_Hal_01_0038 (93 %)	Sediment, ocean drilling core, Shimokita Penninsula, Japan	AB797725.1
798	Parvarchaeota	52	Clone SBYN_2138 (98 %)	Hypersaline mat, Guerrero Negro, Mexico	JN461389.1
768	Parvarchaeota	48	Clone SBYH_2063 (100 %)	Hypersaline mat, Guerrero Negro, Mexico	JN455195.1
820	Parvarchaeota	47	Clone C9001C_B01_2_C034 (94 %)	Sediment, ocean subseafloor, Shimokita Penninsula, Japan	AB645182.1
456	OD1	112	Clone MAT-CR-P5-B08 (99 %)	Hypersaline microbial mat, Candeleria lagoon, Puerto Rico	EU246201.1
418	OD1	96	Clone KZNMV-5-B58 (96 %)	Kazan Mud Volcano, Eastern Mediterranean	FJ712474.1
510	OD1	58	Clone XH57 (94 %)	Salt lake brine, Inner Mongolia, China	FM210874.1
759	OD1	52	Clone OGT_B5_45 (90 %)	Hadopelagic sediments, Ogasawara Trench, Japan	AB583359.1
950	OD1	42	Clone ODP1251AQB1.35 (91 %)	Subseafloor sediments, Cascadia Margin	AB364354.1
301	SAR406	120	Clone ST-12K17 (98 %)	Brine-seawater interface, Shaban Deep, Red Sea	AJ347757.1

B. Fara Fund Lake

OTU*	Classification	Sequence number	Nearest match (% identity)	Source location of the match	GenBank ID
446	Parvarchaeota	133	Clone Exp331_INH_50U_57 (91 %)	Ocean drilling core sediment, Okinawa, Japan	AB825844.1
190	Bacteria	319	Clone GUAY_50enr_Bac50 (94 %	Hydrothermal sediments, Guaymas Basin, Pacific Ocean	FR682656.1
112	Bacteria	256	Clone GeoB15101-6-B5-Bact-49 (96 %)	Urania Basin brine lake, Eastern Mediterranean	KM278882.1
335	Bacteria	86	Clone GeoB15101-6-B5-Bact-49 (95 %)	Urania Basin brine lake, Eastern Mediterranean	KM278882.1
284	Bacteria	83	Clone: p816_b_5.05 (91 %)	Sediment, hydrothermal vent, Okinawa Trough	AB305603.1
388	Bacteria	70	Clone MS-A229 (87 %)	Calcareous sandy sediment, Spain	FJ949402.1
1957	Bacteria	70	Clone LGNSa2_BC_022 (95 %)	Hypersaline mat, Guerrero Negro, Mexico	EU687381.1
646	Bacteria	61	Clone Napoli-4B-82 (91 %)	Napoli mud volcano hypersaline marine sediment	AY592796.1
409	Bacteria	57	Clone MAT-CR-H3-H11 (97 %)	Hypersaline microbial mat, Candeleria lagoon, Puerto Rico	EU245196.1
495	Bacteria	51	Clone 90b (92 %)	Anaerobic reactor fed with effluent from the chemical industry	FJ462088.1
545	Bacteria	51	Clone AS07-7-39 (88 %)	Biofilm from subsurface sulfidic cave stream	KM410335.1
889	Bacteria	51	Clone TVG12-B32 (93 %)	Deep-sea hydrothermal vent sediments, Pacific Ocean	KM071721.1
849	Bacteria	48	Clone NNCTHY2917 (92 %)	Anaerobic UASB reactor fed with domestic sewage	AB941245.1
571	Bacteria	43	Clone SBYZ_1565 (94 %)	Hypersaline mat, Guerrero Negro, Mexico	JN496253.1
165	Bacteria	41	Clone LGNSa2_BC_022 (94 %)	Hypersaline mat, Guerrero Negro, Mexico	EU687381.1
9	Bacteria	41	Clone LGNSa2_BC_022 (100 %)	Hypersaline mat, Guerrero Negro, Mexico	EU687381.1
293	Bacteria	37	Clone TOSC-55 (88 %)	Sediment	LN650518.1
518	Parvarchaeota	95	Clone OGAP_AB16_38cm_27 (91 %)	West Pacific abyssal plain sediments	AB827103.1
227	Parvarchaeota	82	Clone GN21D31B (97 %)	Hypersaline mat, Guerrero Negro, Mexico	EU731482.1
618	Parvarchaeota	70	Clone P66A (95 %)	Evaporitic crust, Guerrero Negro, Mexico	EF106720.1
679	Parvarchaeota	67	Clone A42 (94 %)	Moderate saline soil, China	EU328116.1
652	Parvarchaeota	63	Clone Exp331_INH_65U_08 (92 %)	Ocean drilling core sediment, Okinawa, Japan	AB825870.1

3125	Parvarchaeota	47	Clone P53A (98 %)	Evaporitic crust, Guerrero Negro, Mexico	EF106700.1
906	Parvarchaeota	44	Clone GNA02D12 (98 %)	Hypersaline mat, Guerrero Negro, Mexico	EU731484.1
1371	Parvarchaeota	35	Clone Y17A (97 %)	Hypersaline mat, Guerrero Negro, Mexico	EF106701.1
3987	Parvarchaeota	34	Clone TVG1-A103 (90 %)	Deep-sea hydrothermal vent sediments, East Pacific Ocean	KJ564030.1
90	Parvarchaeota	33	Clone GN21D31B (99 %)	Hypersaline mat, Guerrero Negro, Mexico	EU731482.1
142	SAR406	381	Clone Kasin-B3-C09 (92 %)	Hypersaline sediment, Kalmykian Steppe, Russia	HE604724.1
301	SAR406	118	Clone ST-12K17 (98 %)	Brine-seawater interface, Shaban Deep, Red Sea	AJ347757.1
1911	SAR406	33	Clone ST-12K17 (95 %)	Brine-seawater, Shaban Deep, Red Sea	AJ347757.1
1191	SAR406	33	Clone E10Dbac (99 %)	Great Salt Lake sediment, USA	DQ386205.1
179	OP3	399	Clone MEDEE_16SB_09_UIF (98 %)	Water, Hypersaline Lake Medee, Eastern Mediterranean	JX446112.1
487	OP3	62	Clone SCS_HX36_154 (96 %)	Surface sediment, China Sea	HM598263.1
821	OP3	42	Clone Clip 7 (95 %)	Stratified lagoon, Clipperton Island	HQ691945.1
137	WS3	341	Clone HA9-SRB-CD3 (97 %)	Sediment from hydrothermal vent, Bransfield Strait, Antarctica	FM868270.1
278	WS3	31	Clone SBZP_6337 (100 %)	Hypersaline mat, Guerrero Negro, Mexico	JN539471.1
250	Caldithrix	136	Clone SBYZ_5190 (98 %)	Hypersaline mat, Guerrero Negro, Mexico	JN493439.1
3721	Caldithrix	129	Clone SBYZ_5190 (98 %)	Hypersaline mat, Guerrero Negro, Mexico	JN493439.1
520	Caldithrix	45	Clone SBYZ_5190 (95 %)	Hypersaline mat, Guerrero Negro, Mexico	JN493439.1
454	OD1	80	Clone Kasin-B1-G09 (90 %)	Hypersaline sediment, Kalmykian Steppe, Russia	HE604802.1
1189	OD1	48	Clone HA-EUB-T9-47 (95 %)	Sediment from hydrothermal vent, Bransfield Strait, Antarctica	FM867974.1
850	OD1	45	Clone HA-EUB-T9-47 (89 %)	Sediment from hydrothermal vent, Bransfield Strait, Antarctica	FM867974.1
510	OD1	43	Clone Kasin-B2-D09 (99 %)	Hypersaline sediment, Kalmykian Steppe, Russia	HE604672.1
2433	OD1	36	Clone Kasin-B2-D09 (96 %)	Hypersaline sediment, Kalmykian Steppe, Russia	HE604672.1
307	WWE1	133	Clone 104B378 (98 %)	Sediment, Chefren mud volcano, Eastern Mediterranean	EF687307.1
49	WWE1	82	Clone MAT-CR-H3-B03 (91 %)	Hypersaline microbial mat, Candeleria lagoon, Puerto Rico	EU245152.1
666	OP1	70	Clone Kasin-B2-G04 (100 %)	Hypersaline sediment, Kalmykian Steppe, Russia	HE604693.1

* Only OTUs with abundances above 0.05% are included.

Table S4. Taxonomic composition of sediments from Ursu and Fara Fund lakes (A) and the extended phylum level diversity (B).

A.

Phylum-level taxonomy*

Phylum	Abundance (%)	Lake	Phylum	Abundance (%)	Lake
OD1	1.1	Ursu	*Proteobacteria*	32.4	Ursu
WWE1	1.2	Ursu	SAR406	1.0	Fara Fund
Caldithrix	1.3	Ursu	*Acidobacteria*	1.7	Fara Fund
WS3	1.4	Ursu	*Euryarchaeota*	1.8	Fara Fund
OP1	1.5	Ursu	*Gemmatimonadetes*	1.8	Fara Fund
OP3	1.7	Ursu	*Planctomycetes*	1.9	Fara Fund
Planctomycetes	2.4	Ursu	*Parvarchaeota*	2.2	Fara Fund
Actinobacteria	2.6	Ursu	*Firmicutes*	2.4	Fara Fund
Crenarchaeota	2.7	Ursu	*Spirochaetes*	3.1	Fara Fund
Parvarchaeota	3.6	Ursu	*Crenarchaeota*	5.7	Fara Fund
Euryarchaeota	4.4	Ursu	*Actinobacteria*	7.4	Fara Fund
Spirochaetes	4.8	Ursu	*Chloroflexi*	7.8	Fara Fund
Chloroflexi	8.7	Ursu	*Bacteroidetes*	10.6	Fara Fund
Bacteroidetes	11.6	Ursu	*Proteobacteria*	39.3	Fara Fund

Class-level taxonomy*

Class	Abundance (%)	Lake	Class	Abundance (%)	Lake
Rhodothermi	1.3	Ursu	MBGB	1.6	Fara Fund
Phycisphaerae	1.6	Ursu	*Acidimicrobiia*	1.7	Fara Fund
Saprospirae	2.0	Ursu	*Actinobacteria*	1.7	Fara Fund
MBGB	2.2	Ursu	*Clostridia*	1.8	Fara Fund
Actinobacteria	2.2	Ursu	Parvarchaea	2.2	Fara Fund
Thermoplasmata	3.2	Ursu	*Bacteroidia*	2.5	Fara Fund
Parvarchaea	3.5	Ursu	Ellin6529	2.8	Fara Fund
Spirochaetes	4.0	Ursu	*Spirochaetes*	3.0	Fara Fund
Bacteroidia	5.0	Ursu	*Thermoleophilia*	3.1	Fara Fund
Alphaproteobacteria	6.4	Ursu	*Anaerolinea*	3.1	Fara Fund
Anaerolinea	7.9	Ursu	*Thaumarchaeota*	3.4	Fara Fund
Gammaproteobacteria	8.1	Ursu	*Betaproteobacteria*	4.2	Fara Fund
Deltaproteobacteria	16.6	Ursu	*Gammaproteobacteria*	6.8	Fara Fund
Rhodothermi	1.0	Fara Fund	*Alphaproteobacteria*	12.1	Fara Fund
Phycisphaerae	1.1	Fara Fund	*Deltaproteobacteria*	16.1	Fara Fund
Saprospirae	1.1	Fara Fund			

Order-level taxonomy*

Order	Abundance (%)	Lake	Order	Abundance (%)	Lake
			GCA004	1.2	Fara Fund
MSBL9	1.0	Ursu			
Rhodothermales	1.3	Ursu	*Myxococcales*	1.3	Fara Fund
Thiotrichales	1.4	Ursu	*Burkholderiales*	1.3	Fara Fund
Saprospirales	2.0	Ursu	MND1	1.4	Fara Fund
Chromatiales	2.1	Ursu	OPB11	1.7	Fara Fund
Actinomycetales	2.1	Ursu	*Actinomycetales*	1.7	Fara Fund
Desulfarculales	2.5	Ursu	*Acidimicrobiales*	1.7	Fara Fund
Desulfovibrionales	2.6	Ursu	*Rhodobacterales*	1.7	Fara Fund

E2	3.2	Ursu	*Desulfarculales*	1.9	Fara Fund
Rhodobacterales	3.5	Ursu	*Sphingomonadales*	2.2	Fara Fund
Spirochaetales	4.0	Ursu	*Gaiellales*	2.3	Fara Fund
Bacteroidales	5.0	Ursu	*Desulfuromonadales*	2.5	Fara Fund
OPB11	6.2	Ursu	*Bacteroidales*	2.5	Fara Fund
Desulfobacterales	8.4	Ursu	*Spirochaetales*	3.1	Fara Fund
Halanaerobiales	1.0	Fara Fund	*Xanthomonadales*	3.3	Fara Fund
Rhodothermales	1.0	Fara Fund	*Nitrososphaerales*	3.4	Fara Fund
Desulfovibrionales	1.0	Fara Fund	*Rhizobiales*	6.2	Fara Fund
Saprospirales	1.1	Fara Fund	*Desulfobacterales*	8.1	Fara Fund

Family-level taxonomy*

Family	Abundance (%)	Lake	Family	Abundance (%)	Lake
Balneolaceae	1.3	Ursu	*Comamonadaceae*	1.3	Fara Fund
Piscirickettsiaceae	1.4	Ursu	*Rhodobacteraceae*	1.6	Fara Fund
Saprospiraceae	2.0	Ursu	*Desulfarculaceae*	1.9	Fara Fund
Desulfohalobiaceae	2.4	Ursu	*Sphingomonadaceae*	1.9	Fara Fund
Desulfarculaceae	2.5	Ursu	*Gaiellaceae*	2.3	Fara Fund
Rhodobacteraceae	3.1	Ursu	*Sinobacteraceae*	2.7	Fara Fund
Spirochaetaceae	4.0	Ursu	*Spirochaetaceae*	3.0	Fara Fund
Desulfobacteraceae	8.3	Ursu	*Nitrososphaeraceae*	3.4	Fara Fund
Halanaerobiaceae	1.0	Fara Fund	*Hyphomicrobiaceae*	4.4	Fara Fund
Desulfohalobiaceae	1.0	Fara Fund			

Genus-level taxonomy*

Genus	Abundance (%)	Lake	Genus	Abundance (%)	Lake
KSA1	1.2	Ursu	*Spirochaeta*	1.8	Fara Fund
Desulfotignum	1.4	Ursu	*Rhodoplanes*	2.8	Fara Fund
Desulfovermiculus	1.7	Ursu	*Candidatus* Nitrososphaera	3.4	Fara Fund
Spirochaeta	1.8	Ursu			

*Only taxa with abundances ≥ 1% are presented.

B.

Domain	Phylum	No of OTUs	No of reads		% of total reads	
			Ursu Lake	Fara Fund Lake	Ursu Lake	Fara Fund Lake
Archaea	Parvarchaeota	231	2988	1406	3.55	2.23
Archaea	Crenarchaeota	33	2293	3566	2.73	5.65
Archaea	Euryarchaeota	107	3675	1113	4.37	1.76
Bacteria	Caldithrix	22	1081	339	1.28	0.54
Bacteria	Thermi	5	780	53	0.93	0.08
Bacteria	AC1	8	199	68	0.24	0.11
Bacteria	Acidobacteria	69	316	1082	0.38	1.71
Bacteria	Actinobacteria	198	2198	4646	2.61	7.36
Bacteria	Armatimonadetes	2	9	5	0.01	0.01
Bacteria	Bacteroidetes	248	9750	6708	11.59	10.63
Bacteria	BHI80-139	1	0	4	0.00	0.01
Bacteria	BRC1	18	132	43	0.16	0.07
Bacteria	Chlamydiae	19	100	82	0.12	0.13
Bacteria	Chlorobi	18	517	38	0.61	0.06
Bacteria	Chloroflexi	171	7346	4914	8.73	7.79
Bacteria	Cyanobacteria	8	356	360	0.42	0.57
Bacteria	Deferribacteres	3	48	15	0.06	0.02
Bacteria	Elusimicrobia	9	62	62	0.07	0.10
Bacteria	FCPU426	1	10	0	0.01	0.00
Bacteria	FBP	1	0	3	0.00	0.00

Bacteria	Fibrobacteres	6	75	19	0.09	0.03
Bacteria	Firmicutes	96	486	1505	0.58	2.39
Bacteria	Fusobacteria	4	8	18	0.01	0.03
Bacteria	GAL15	1	0	3	0.00	0.00
Bacteria	Gemmatimonadetes	42	641	1116	0.76	1.77
Bacteria	GN02	9	96	18	0.11	0.03
Bacteria	GN04	17	356	13	0.42	0.02
Bacteria	H-178	3	176	0	0.21	0.00
Bacteria	Hyd24-12	5	221	72	0.26	0.11
Bacteria	KSB3	6	276	11	0.33	0.02
Bacteria	LCP-89	5	82	26	0.10	0.04
Bacteria	Lentisphaerae	24	724	73	0.86	0.12
Bacteria	Nitrospirae	8	0	202	0.00	0.32
Bacteria	NKB19	17	202	15	0.24	0.02
Bacteria	OD1	79	908	536	1.08	0.85
Bacteria	OP1	17	1286	167	1.53	0.26
Bacteria	OP11	8	143	17	0.17	0.03
Bacteria	OP3	51	1402	623	1.67	0.99
Bacteria	OP8	9	673	88	0.80	0.14
Bacteria	OP9	3	251	491	0.30	0.78
Bacteria	PAUC34f	3	58	0	0.07	0.00
Bacteria	Planctomycetes	227	2017	1195	2.40	1.89
Bacteria	Proteobacteria	858	27270	24794	32.42	39.29
Bacteria	SAR406	12	251	644	0.30	1.02
Bacteria	SBR1093	1	120	0	0.14	0.00
Bacteria	Spirochaetes	95	4074	1967	4.84	3.12
Bacteria	SR1	3	35	3	0.04	0.00
Bacteria	Synergistetes	4	53	24	0.06	0.04
Bacteria	TA06	1	5	0	0.01	0.00
Bacteria	Tenericutes	7	45	45	0.05	0.07
Bacteria	Thermotogae	4	738	254	0.88	0.40
Bacteria	TM6	26	187	98	0.22	0.16
Bacteria	TM7	9	34	31	0.04	0.05
Bacteria	Verrucomicrobia	60	593	538	0.70	0.85
Bacteria	WS1	11	133	75	0.16	0.12
Bacteria	WS2	3	27	0	0.03	0.00
Bacteria	WS3	41	1158	552	1.38	0.87
Bacteria	WWE1	17	1020	222	1.21	0.35
Bacteria	ZB3	2	35	0	0.04	0.00

Supplementary figures

Figure S1

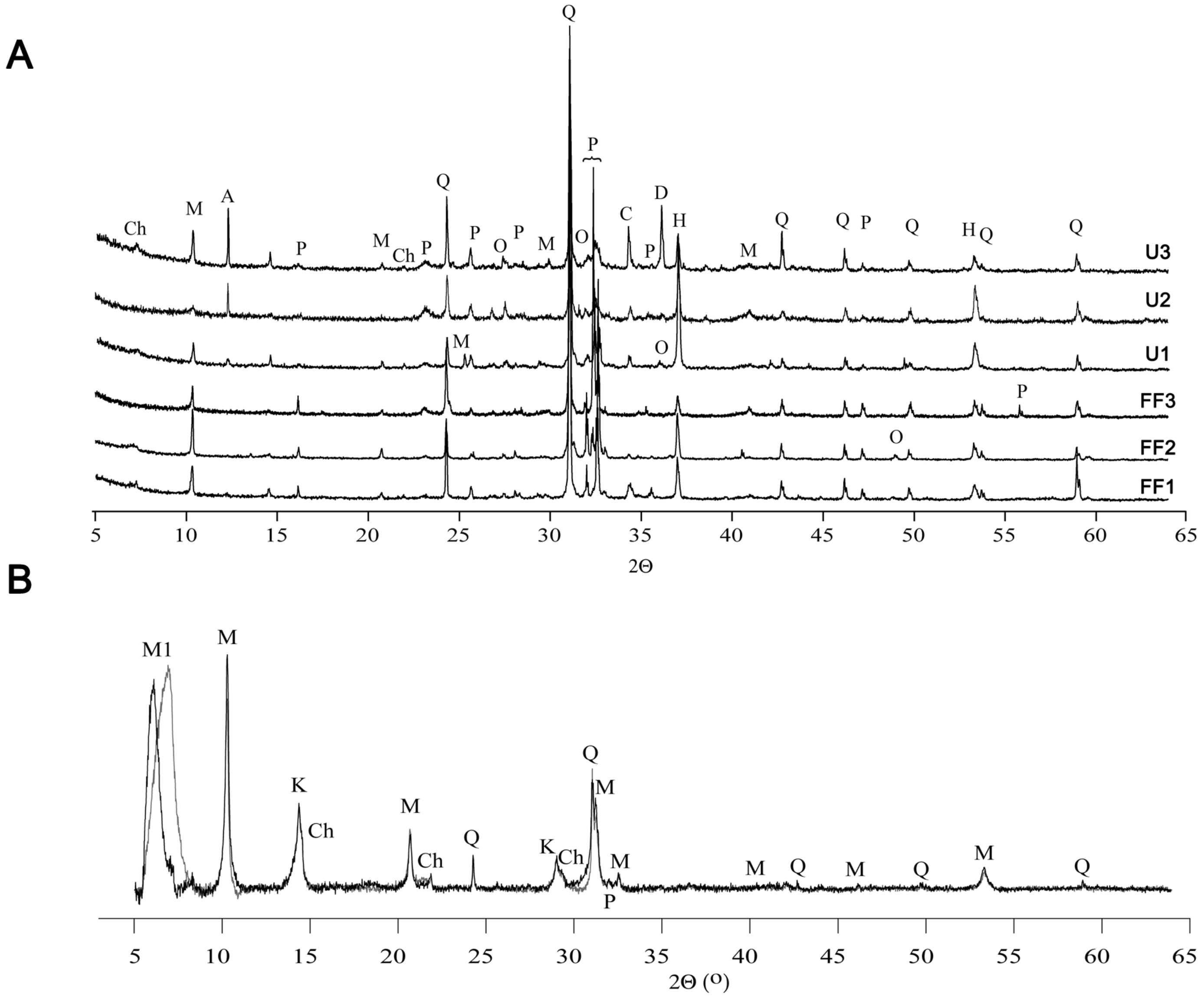

(**A**) Mineral composition of Ursu (U) and Fara Fund (FF) sapropels from three distinct sediment samples. (Q - quartz, P – plagioclase feldspar, O – orthoclase feldspar, M – muscovite, Ch – chlorite, A – amphibole, C – calcite, D – dolomite, H – halite). (**B)** X-ray diffractions on the <2μm size fraction separated from the sapropels (gray – oriented sample; black – sample treated with ethylene glycol): M1 – montmorillonite, M – muscovite, K – Kaolinite, Ch – chlorite; small amounts of quartz (Q) and plagioclase feldspar (P) are also present in the samples.

Figure S2

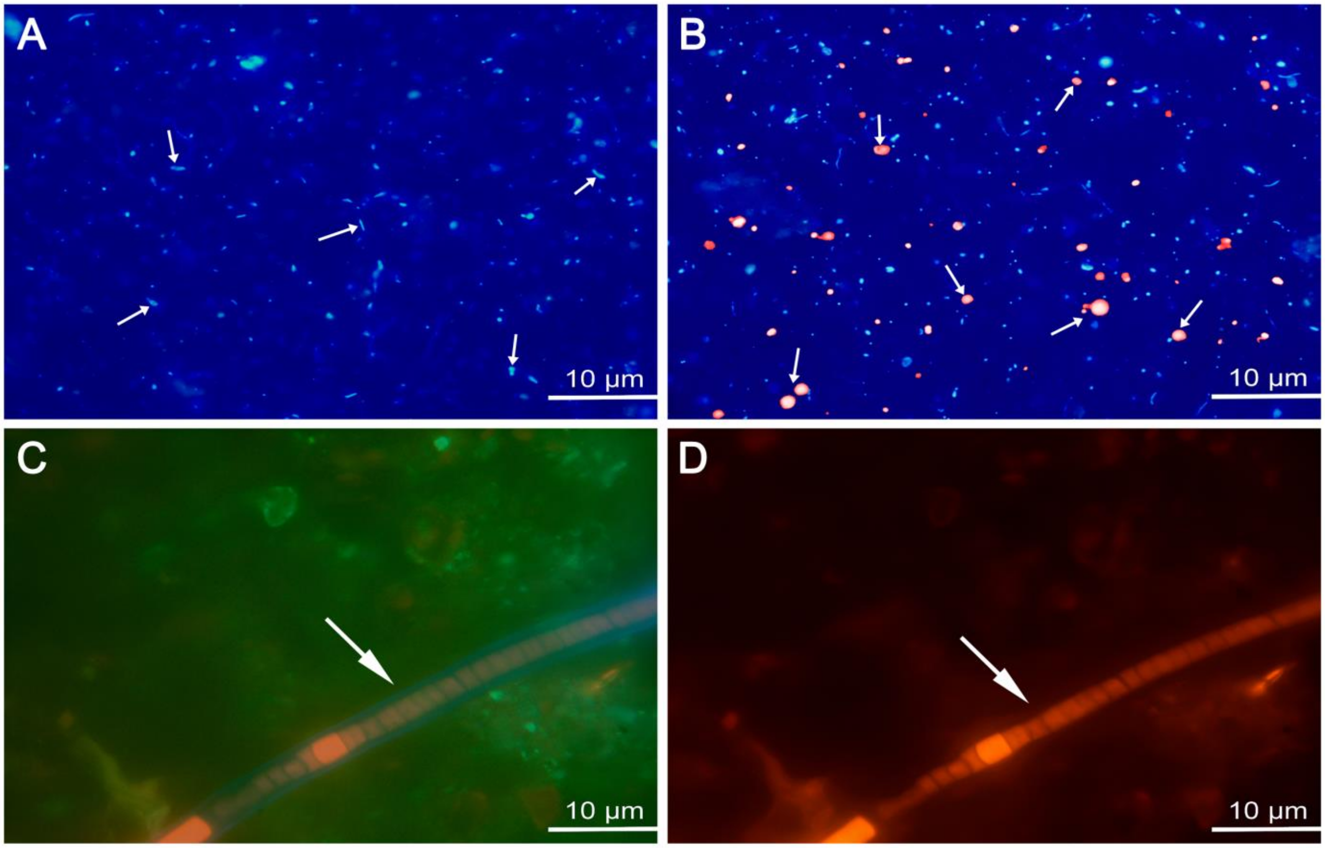

Epifluorescence microscopy images from Ursu and Fara Fund sapropels. (A) epifluorescence DAPI image of prokaryotic morphotypes (indicated by arrows) in Fara Fund Lake sapropel. (B) superimposed image of Ursu Lake sapropel showing the presence of chlorophyll-containing microorganisms (indicated by arrows). (C) and (D) show the presence of filamentous photosynthetic bacteria in Ursu Lake sapropel (indicated by arrows).

Figure S3

Habitat distribution of the Ursu and Fara Fund sapropels' OTUs (>0.1% abundance) closest blastn hits, as found in the NCBI's nucleotide database.

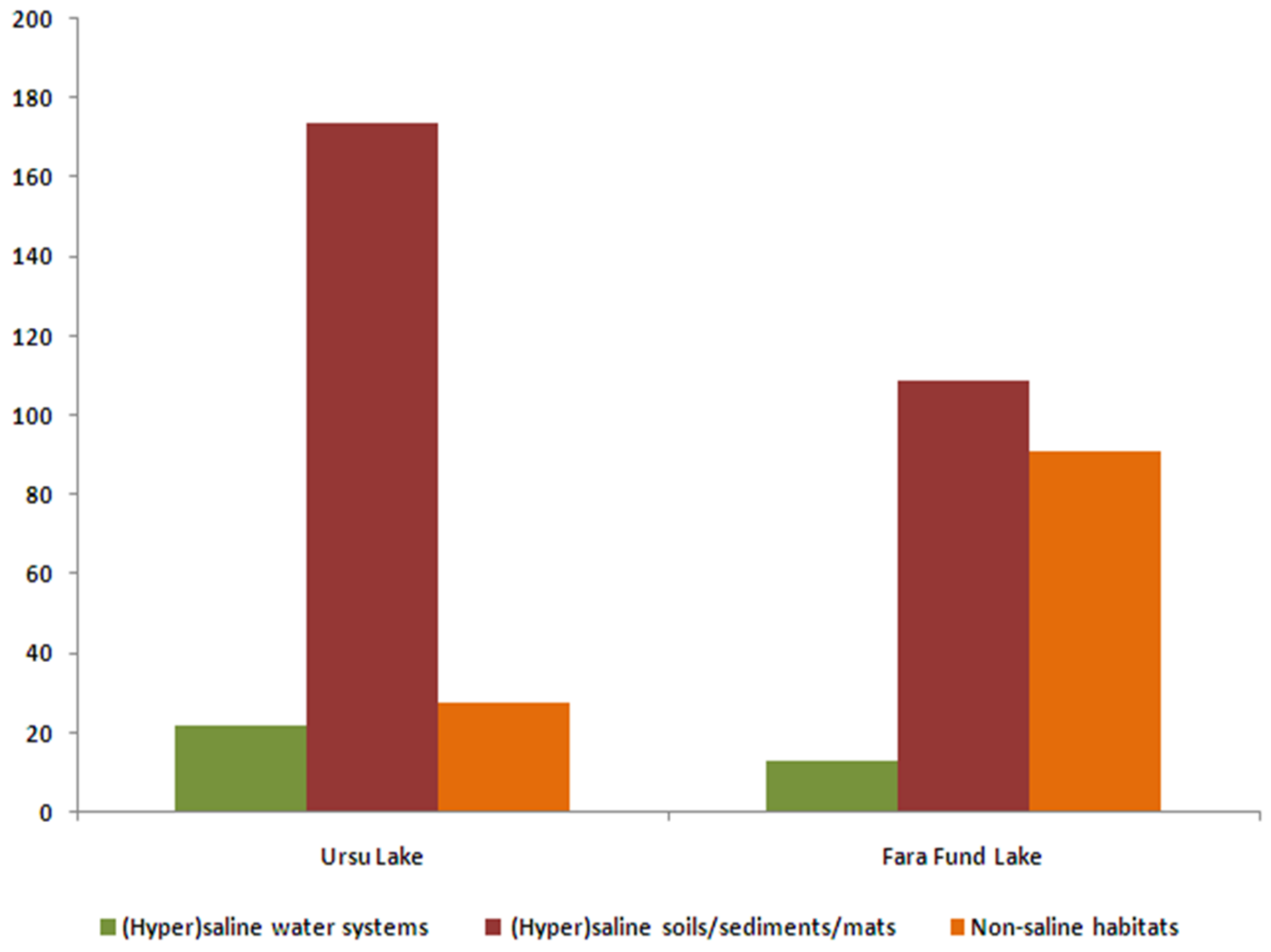

Figure S4

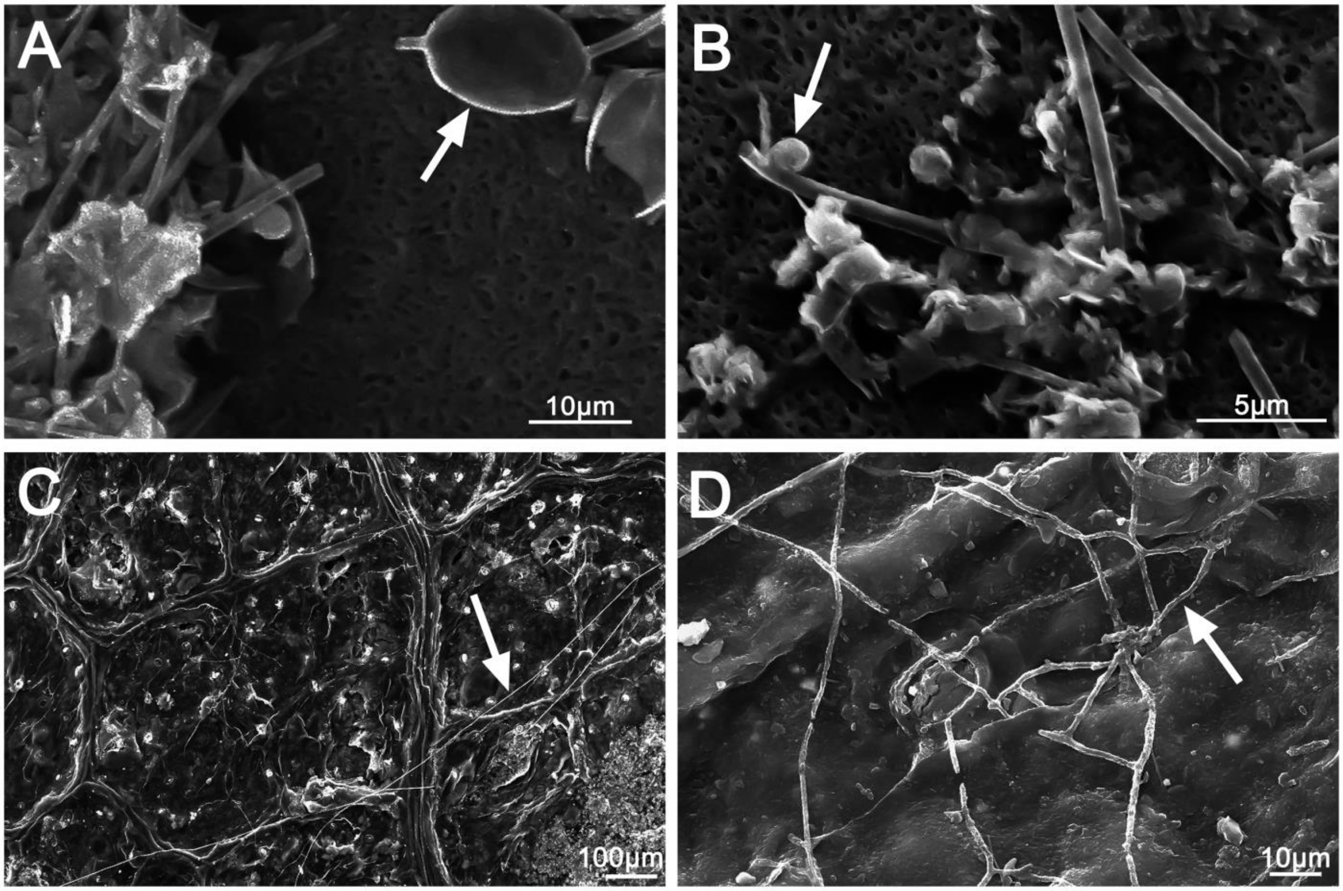

SEM images of fungal morphotypes (indicated by arrows) present in the sapropel samples (A and B) and on the surface of plant material recovered from sapropels (C and D).

Figure S5

(**A**) Dendrogram constructed using the fungal community fingerprints generated from twelve environmental samples. (**B**) Fungal phylogenetic tree created using the V7-V8 SSU rDNA hypervariable regions. Legend: L-lane; B-band.

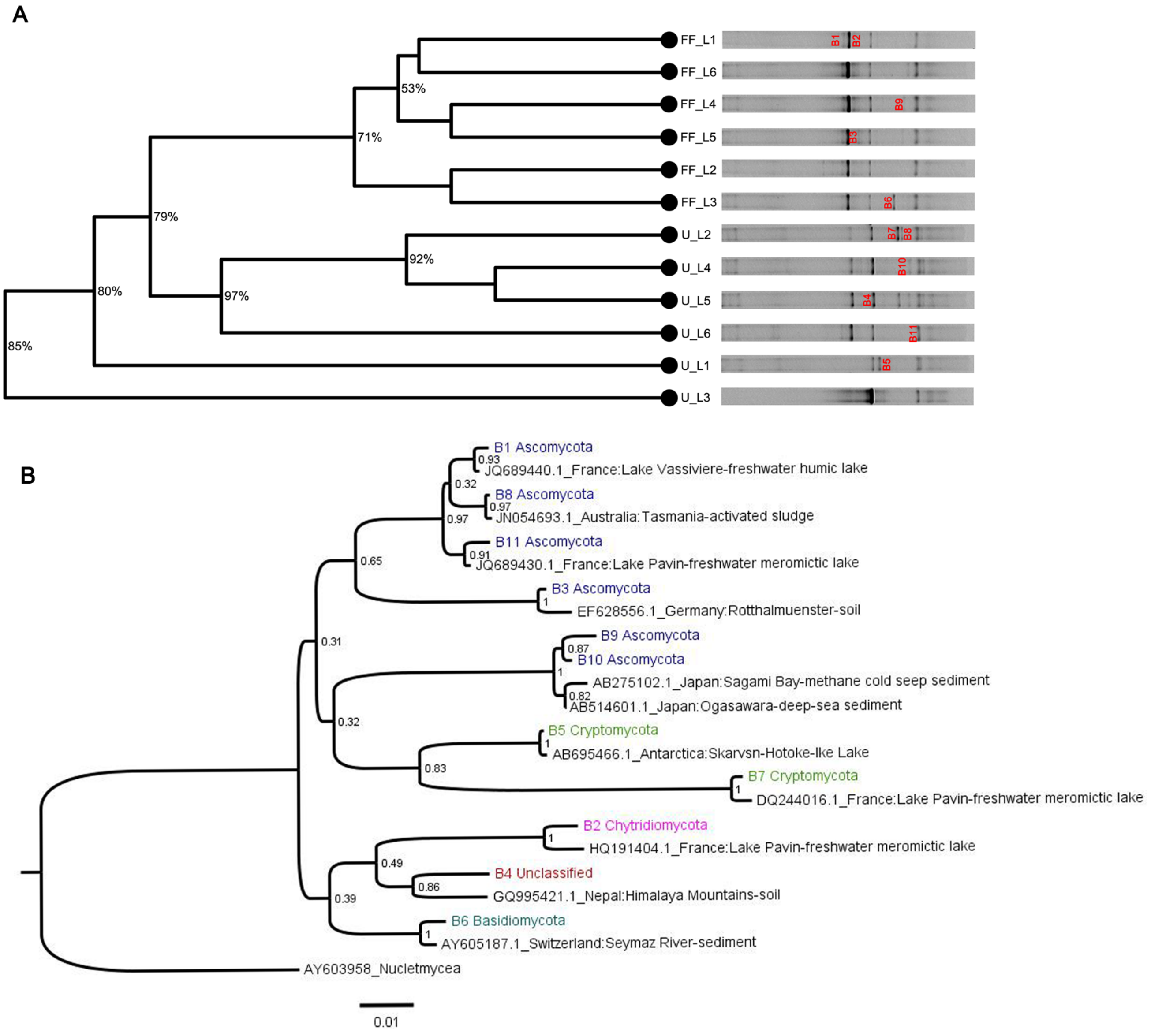

Supplement: Supplementary file 1 — Supplementary Information [file 41598_2017_6232_MOESM1_ESM.pdf]
